# Endocrine and Electrolyte Balances during Periovulatory Period in Cycling Mares

**DOI:** 10.3390/ani11020520

**Published:** 2021-02-17

**Authors:** Katiuska Satué, Esterina Fazio, Ana Muñoz, Pietro Medica

**Affiliations:** 1 Department of Animal Medicine and Surgery, Faculty of Veterinary Medicine, CEU-Cardenal Herrera University, 46115 Valencia, Spain; 2 Department of Veterinary Sciences, Veterinary Physiology Unit, Polo Universitario Annunziata, Viale Palatucci 13, 98168 Messina, Italy; fazio@unime.it (E.F.); pmedica@unime.it (P.M.); 3 Department of Animal Medicine and Surgery, School of Veterinary Medicine, University of Córdoba, 14014 Córdoba, Spain; pv1mujua@uco.es

**Keywords:** ACTH, aldosterone, cortisol, cycling mare, electrolytes

## Abstract

**Simple Summary:**

This study provides new evidence on the physiological mechanisms involved in the electrolyte balance during periovulatory period in cycling mares. The interrelationships among adrenocorticotropic hormone (ACTH), cortisol (CORT), aldosterone (ALD) and electrolytes (sodium—Na^+^, potassium—K^+^ and chloride—Cl^−^) were evaluated. The simultaneous increase in ACTH, CORT and ALD toward the time of ovulation could suggest the involvement of the adrenocortical pituitary axis in the ovulatory mechanisms, contributing at the same time to the maintenance of electrolyte homeostasis.

**Abstract:**

In cycling females, the periovulatory period is characterized by stimulation of the hypothalamic pituitary adrenal (HPA) axis. The aim of present study was to analyze the pattern and interrelationships among adrenocorticotropic hormone (ACTH), cortisol (CORT), aldosterone (ALD) and electrolytes (sodium—Na^+^, potassium—K^+^ and chloride—Cl^−^) during periovulatory period in cycling mares. Venous blood samples were obtained daily from a total of 23 Purebred Spanish broodmares, aged 7.09 ± 2.5 years, from day −5 to day +5 of estrous cycle, considering day 0, the day of ovulation. Plasma ACTH was measured by a fluorescent immunoassay kit, serum CORT and ALD by means of a competitive ELISA immunoassay, and plasma Na^+^, K^+^ and Cl^−^ were quantified by an analyzer with selective electrodes for the three ions. ACTH showed higher concentrations at day 0 compared to days −5 to −1 and +1 to +3 (*p* < 0.05). CORT showed higher concentrations at day 0 compared to days −5 to −2 and +1 to +5 (*p* < 0.05). ALD showed higher concentrations at day 0 compared to days −5 to −2 (*p* < 0.05) and +2 (*p* < 0.05). Na^+^ and Cl^−^ showed higher concentrations at day 0, compared to day −5 and +5. K^+^ showed lower concentrations at day 0 compared to day +1 (*p* < 0.05). The significant correlations obtained between ACTH and CORT (r = 0.20) and between ACTH and ALD (r = 0.32) suggest that although ACTH may have an effect both on CORT and ALD, there are other very important determinants that could be considered. Hence, it is possible to presume that the pituitary adrenocortical response and ALD may be involved in the ovulatory mechanisms without a direct relation with electrolyte pattern.

## 1. Introduction

In cycling mares, studies on the hypothalamic pituitary adrenal (HPA) axis response to stress under different physiological conditions such, transport [1], and gynecological manipulations for artificial insemination [2], pregnancy [3], weaning [4], nutritional imbalances [5], exercise [6], pain [7], and social stress [8] have been widely documented. Stress, inducing an increase of adrenocorticotropic pituitary hormone (ACTH) and cortisol (CORT) responses, exerts negative effects on reproduction; in fact, it suppresses the release of gonadotropin-releasing hormone (GnRH), interferencing with the pulsatility and the peak of luteinizing hormone (LH) and changing the secretion of oestradiol-17β (E2) with a decrease of the sexual behavior, ovulation, and fertility [9]. The baseline ACTH concentrations in cyclic mares in non-stressful conditions are unknown, and the scarce evidence on CORT is contradictory, such as an increase along luteal [10,11] and ovulatory periods [12,13].

On the other hand, the activation of renin angiotensin aldosterone system (RAAS) during periovulatory period in mare [14] and its progressive increases, as soon as the significant positive correlations between estrogens 17-β (E2) and ALD [15] and between aldosterone (ALD) and follicle diameter, have been documented [16]. However, the possible relationships among ACTH, CORT, and ALD and electrolytes (sodium—Na^+^, potassium—K^+^ and chloride—Cl^−^) in the mare in ovulatory period have not been clearly established.

ALD exerts differential effects in relation to estrous cycle, since it is related to folliculogenesis, oocyte maturation, ovulation, development of the corpus luteum (CL), and steroidogenesis [17]. On the one hand, ALD increases during luteal period in women with high but not low sodium balance [18] and in mares [14,16] according to the P4 secretion [18]. On the other hand, the ovulatory peak of ALD in women is related to the stimulatory effect of estrogen on the synthesis of angiotensinogen (AOGEN) [19], with modifications of Na^+^ concentrations at the level of the dense macula, and alterations in local sympathetic activity [20].

In mares, Kinslow et al. [21] showed large increases in the urinary excretion of Na^+^ in samples during the period from day −2 to + 4 days of estrus, compared to the rest of the cycle. P4 is generally considered to be a “Na^+^—losing” steroid or an “ALD antagonist” [21].

Since ACTH and CORT are factors related with ALD synthesis [5,9,22], and ALD with electrolyte equilibrium, possible interactions between pituitary ACTH, adrenal steroids and electrolytes during estrous cycle could be hypothesized. The aim of present study was to analyse the pattern and interrelationships among ACTH, CORT, ALD, and electrolytes: Na^+^, K^+^ and Cl^−^ during periovulatory period in cycling mares.

## 2. Materials and Methods

### 2.1. Animals

All methods and procedures used in this study were in compliance with the EU directive (2010/63/EU) on the protection of animals used for scientific purposes. The Animal Ethics Committee for the Care and Use of Animals of the CEU-Cardenal Herrera University (Valencia, Spain) concluded that the proposed study did not need ethical approval, as it did not qualify as an animal experiment under Spanish law. This study was carried out in spring, during the months of March and April in 2019. The study was carried out in 23 healthy mares, aged 7.09 ± 2.5 years. The inclusion criteria for the animals were: (1) absence of reproductive diseases in the clinical examination; (2) absence of inflammatory processes or infections that had required treatment or hospitalization during the month prior to the onset of the study; (3) to be vaccinated and dewormed correctly and (4) to be younger than 15-years old, to have no conformation defects that affect the perineum and vulva and lack of previous history of reproductive diseases that affect fertility. All animals were subjected to the same conditions of management, feeding, and reproductive control. The mares were housed in individual stalls and received a diet composed of 4 kg/day of mixed grains and 3 kg/day of commercial horse concentrate, divided into two intakes in the morning and evening; an aliquot of fresh cut grass was added once a day. Water was provided ad libitum and the animals had access to a mineral block during the study period.

### 2.2. Reproductive Monitoring of the Mares

Follicular development was checked by rectal ultrasound examination (Ultrasound: Sonosite 180 Plus; SonoSite Inc., Bothell, WA, USA) of mares when they show signs of estrus until the time of ovulation. After ovulation, daily echographic exams were also performed to confirm the development and maturation of the CL, up to 5 days after ovulation. Hormonal treatments in order to synchronize reproductive cycles were not used and, therefore, only natural cycles were included in the current research.

### 2.3. Venous Blood Sampling

Because the objective of the current study was to establish the relationships among ACTH, CORT and ALD during the periovulatory period, blood samples were taken every day, from day −5 to day +5 of ovulation. To reduce the influence of daily rhythms on the release of hormones, all blood samples were collected between 9.00 and 10.30 am, always by the same operator, with the mares at rest and before they received the grain ration. Blood samples were collected from jugular vein using 30 cc syringes, and were divided into three fractions of similar volumes and immediately transferred to: (a) polypropylene tube containing EDTA (1 mg/mL of blood), (b) lithium heparin (12–30 UI/mL) and (c) glass tubes without anticoagulant (Tapval^®^, Barcelona, Spain). Samples were centrifuged at 3000× *g* during 10 min at 4 °C, plasma and serum was harvested and stored at −20 °C until analysis.

### 2.4. Measurements of Hormone Concentrations

Plasma ACTH concentrations (pg/mL) were measured with a commercial competitive streptavidin-biotin immunoenzymatic technique ACTH Fluorescent Immunoassay Kit (FEK-001-01; Phoenix Pharmaceutical Inc., Burlingame, CA, USA) validated for equine serum samples. The detection limit of the technic was of 9.6 pg/mL. The intra and inter-assay coefficients of variation (CVs) were <10% and <15%, respectively. The technique shows cross-reactivity with ACTH (100%), β-endorphin (1%), corticotrophin releasing factor CRF (0%), alpha-melanocyte stimulating hormone MSH (0%), metenkephalin (0%), alpha atrial natriuretic peptide ANP (0%) and brain natriuretic peptide BNP-32 (0%).

Serum CORT concentrations (ng/mL) were determined by ELISA of competition using the C97 polyclonal antibody validated and characterized in Laboratory of Endocrinology, Department of Physiology, Complutense University of Madrid (Madrid, Spain). This laboratory procedure shows high specificity for CORT, although it has cross-reactivity with prednisolone (15.71%), prednisone (18.9%), cortisone (10.8%), corticosterone (6.4%), 11-deoxycortisol (40.31%), 21-deoxycortisol (5.31%) and dexamethasone (0.1%). None of the mares in the present study received corticoids during the experiment. The sensitivity of the technique was 3 pg/100 mL. The intra-assay and inter-assay CVs were between 3.7–6.63% and 3.92–9.93%, respectively. The recovery percentage of known amounts of the sample was 95%.

Serum ALD (ng/mL) concentrations were measured with a competitive ELISA using polyclonal antibody AD97 and the combination ALD 3CMO-HRP validated and characterized in Laboratory of Endocrinology, Department of Physiology, Complutense University of Madrid (Madrid, Spain). This test showed high specificity for ALD (percentage recovery = 97.6%). The sensitivity of this technique was 15 pg/mL. Intra- and inter-assays CVs were 4.7–6.4% and 8.5–9.6%, respectively. These methods were validated for horse and have been used in previous research in the same species [3,13,14,15,16,23,24].

Plasma Na^+^ (mmol/L), K^+^ (mmol/L) and Cl^−^ (mmol/L) concentrations were quantified by an analyzer with electrodes selective for the three ions (Vetlyte^®^ IDEXX Laboratories Inc., Westbrook, Maine, USA). These determinations were based on the comparison between the sample and a reference electrode, with known concentrations of said electrolytes.

### 2.5. Statistical Analyses

The statistical analysis was made with the Statistica 12.0 Windows program (Statistica Inc., Chapel Hill, NC, USA). Data are presented as mean ± SD. Normality was checked with the Kolmogorov-Smirnov test and the studied variables followed a Gaussian distribution. Differences between the days were assessed by ANOVA for repeated measurements. When statistical significance was reached, a post-hoc analysis was made (Tukey’s test) in order to evaluate significant differences between pre- and post-ovulation days. Pearson’s correlation coefficients (r) were used to measure the relationships, in cycling mares, between all parameters. Differences were considered significant at *p* < 0.05.

## 3. Results

**Figure 1:**
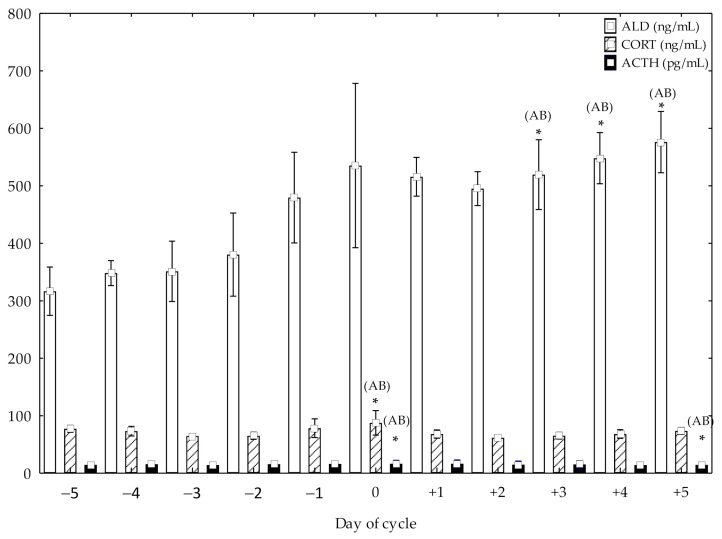
Adrenocorticotrophic hormone (ACTH), cortisol (CORT) and aldosterone (ALD) concentrations (mean ± SD) in cycling mares (n = 23) from day −5 pre-ovulation to day 5 post-ovulation. * Significant differences (A: vs. pre-ovulation days (*p* < 0.05); B: vs post-ovulation days (*p* < 0.05)).

ACTH showed higher concentrations at day 0 compared to pre-ovulation days from −5 to −1 (*p* < 0.05) and post-ovulation days, from +1 to +3 (*p* < 0.05). Higher ACTH concentrations at +5 post-ovulation day compared to pre-ovulation days, from −5 to −1 (*p* < 0.05) and post-ovulation days, from +1 to +4 (*p* < 0.05) were also observed (Figure 1). CORT showed higher concentrations at day 0 compared to pre-ovulation days, from −5 to −2 (*p* < 0.05) and post-ovulation days, from +1 to +5 (*p* < 0.05) (Figure 1). Higher ALD concentrations at +5, +4 and +3 post-ovulation days compared to pre-ovulation days, from −5 to −1 (*p* < 0.05) and post-ovulation days, from +1 to +2 (*p*< 0.05) were also observed (Figure 1).

Na^+^ and Cl^−^ showed higher concentrations at day 0, compared to pre-ovulation day −4 (*p* < 0.05) and post-ovulation day +5 (*p* < 0.05). K^+^ showed higher concentrations at day +1 compared to day −1 (*p* < 0.05) (Table 1).

**Figure 2:**
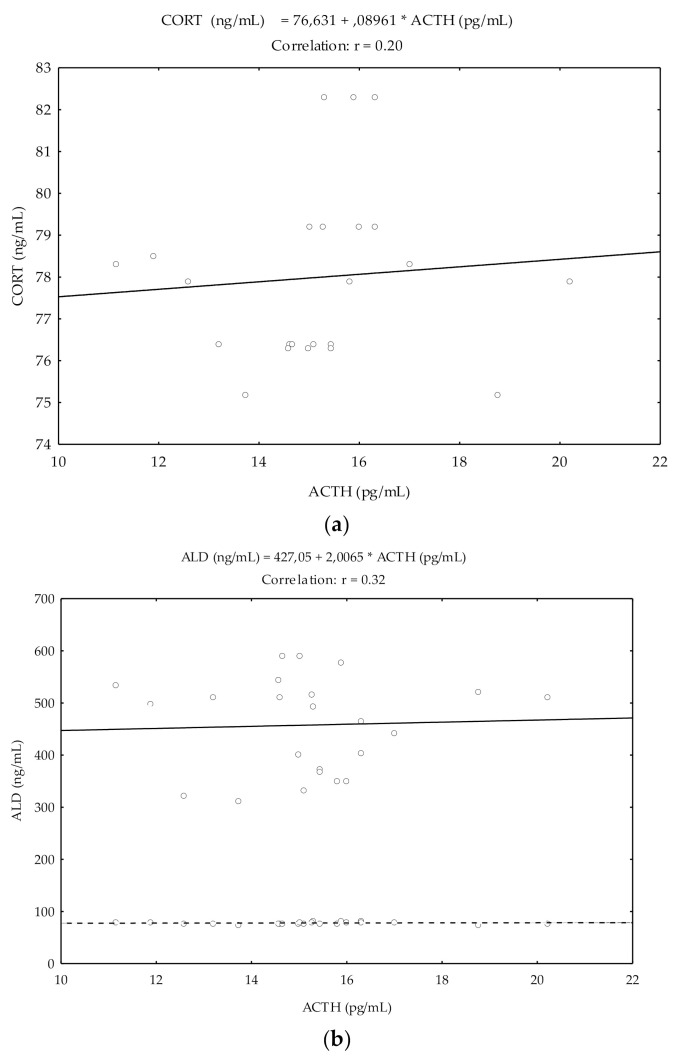
Pearson’s correlation coefficients between ACTH and CORT and between ACTH and ALD in cycling mares (n = 23) from day −5 pre-ovulation to day 5 post-ovulation. Correlation coefficients between ACTH and CORT (**a**) and between ACTH and ALD (**b**).

Pearson’s correlation coefficients (r) showed a significant and positive relationship between ACTH and CORT (r = 0.20; *p* < 0.05), and ACTH and ALD (r = 0.32; *p* < 0.05) (Figure 2).

## 4. Discussion

The concentrations of ACTH and CORT showed similar biphasic patterns; the first highest hormonal peak occurred on day of ovulation and the second at day +5. Although ALD concentrations significantly increased at the time of ovulation, simultaneously to ACTH and CORT, they continued increasing significantly reaching maximum values on day +5. The concentrations of CORT in Spanish Purebred mares confirm those obtained previously, showing an ovulatory CORT peak of 82.00 ng/mL [12], slightly lower than that obtained in this study (87.68 ng/mL), with similarity also in the follicular size in both studies, but different from that previously obtained in ponies [10] and mares [11]. Therefore, while CORT decreases from the beginning of the deviation until the time of luteolysis [11], the maximum concentrations were reached on the day 0 in the present studies. In Spanish Purebred mare, the increase of ACTH and CORT could indicate some degree of participation of HPA axis in follicular development [2].

### 4.1. ACTH and CORT Effects on the Ovulatory Dynamics in Different Species and in Mare

Since glucocorticoids play a modulating role in inflammatory processes [25,26] and accompany to the development of multiple follicular waves and ovulation during estrous it is possible to presume that CORT could be related with the ovulatory peak in mares [27]. Ovulation is triggered by the luteinizing hormone (LH) surge, which initiates a cascade of events in granulosa cells (GCs) initiating luteinization, signaling the egg to commence meiotic maturation, and leading to rupture of the follicle wall. The induction effect of follicle stimulating hormone (FSH) on GCs development facilitated this response to LH. Hence, in mares, LH surge induces 11β-hydroxysteroid dehydrogenase Type 1 (11β-HSD1) and down regulates (11β-HSD2) expression in GCs of the preovulatory follicle [28]. Since 11β-HSD1 converts cortisone to CORT and 11βHSD2 inactivates CORT to cortisone, the change of LH favors the local accumulation of the anti-inflammatory CORT at a time when rapid healing of the ruptured surface is required to restore rapidly normal ovarian function [28].

However, the scientific evidence that supports these events is contradictory, when considering the different species. Therefore, cycling rats exhibit increased HPA activity and higher circulating ACTH and CORT concentrations in proestrus and estrous than metestrous and diestrous [29]. In women, while ACTH secretion shows a cyclic pattern, with low levels during the late follicular phase (days −3 and −2), peak values at days −1 and 0, and a relatively high level during the luteal phase, CORT increases during preovulatory period [30]. Ovulatory peak of CORT in other species, as human [31], sheep [32] and elephant [33] has been documented. In addition, in female dogs, the highest ACTH and CORT concentrations, during proestrus and estrus, were respectively shown [34].

In these studies, no clear correlations were observed between CORT and ACTH in the pre-ovulatory period. On the other hand, in the present study, the sensible but significant correlation between ACTH and CORT could suggest that although there is a close temporal dynamism between both peaks, no proportionality between the serum levels of each occurred.

### 4.2. Effect of Estrous Cycle on the ACTH and CORT Patterns in Different Species

In general, the E2 during estrous cycle could exert a stimulatory effect on the hypothalamus, the corticotropic cells or the adrenal gland cortex, by stimulating corticotropin-releasing hormone (CRH) secretion or inhibiting glucocorticoid feedback, or stimulating ACTH synthesis or CORT synthesis/secretion [35,36]. In rat models and cell culture, E2 increases pro-opiomelanocortin (POMC) mRNA [37] and acts upon the paraventricular nucleus, stimulating the release of CRH [36,38]. POMC is a precursor that contains the sequences of several bioactive peptides including circulating melanocyte stimulating hormone (α-MSH), ACTH and β-endorphin [39]. Also, in women, the estrogens participate in the synthesis of CORT by direct stimulatory effect on the cortisol binding globulin (CBG) [40]. It is possible that E2 concentrations in ovulatory period in the mare have an impact on the HPA axis, stimulating the synthesis and/or secretion of ACTH, by means of different molecular pathways, as occur in humans and experimental animals [35], sheep [41] and female dogs [34].

Two recent studies conducted by the same authors in Spanish Purebred mares showed the highest concentrations of E2 at the time of ovulation [15] and positive correlations between follicular diameter and E2 [16]. Although in mares it is not well known how ovarian steroid hormones affect the HPA axis, the study of adrenal sex hormone receptors is of interest, since their presence or absence indicates whether the ovarian hormone fluctuations occurring during the estrous cycle. However, Hedberg et al. [42,43] were not able to detect any effect of endogenous E2 on the quantity of adrenal steroid hormones produced when intact mares in estrus and after ovariectomy were treated with a synthetic ACTH (tetracosactide). Likewise, the basal CORT pattern differed between intact and ovariectomized mares, suggesting that E2 may affect the adrenal function. Indeed, Alm et al. [44] showed E2 receptors (ER) in cortex and adrenal medulla in ovariectomized mares, showing that the ovulatory dynamics could be the result in the same way of ACTH and CORT effects. Hence, it is possible to suggest the interaction among pre-ovulatory follicle, hypophysis, and adrenal synthesis during the ovulatory period.

### 4.3. Effect of Estrous Cycle on the HPA Axis and ALD in Different Species and in Mare

In rat model, P4 inhibits the sensitizing effects of estrogen on ACTH, with a decrease of ACTH levels according to the P4 increase in estrous and diestrus phases [35,36]. Similar changes were observed by Gallelli et al. [34] in female dogs and in elephants [33]. Carey et al. [45] proposed that P4 may have different stimulatory and inhibitory effects on the HPA axis in the rat, by means of different mechanisms. In Spanish purebred mares, correlations between CORT and P4 suggested certain stimulation of CORT from P4 [16], since P4 is a precursor to CORT, both hormones can be released when adrenal gland is stimulated.

The P4 may also affect ALD levels in several direct or indirect ways. In fact, P4 (1) is a natural precursor of ALD; (2) it stimulates ALD secretion in vitro and in vivo (3) [18,46]; it is a mineralocorticoid receptor (MR) antagonist; (4) it has a direct natriuretic effect [18] that in turn may indirectly stimulate ALD secretion. Finally, LH induces the ALD increase in the luteal phase, the P4 secretion in the ovary, and may also stimulate ALD synthesis in the adrenal cortex [47]. Then, one possible explanation for the higher ALD levels could be a direct stimulatory effect of increased P4 levels, during luteal phase, on ALD production by the zona glomerulosa, occurring independently of the renin angiotensin system (SRA), as proposed by Szmuilowicz et al. [18]. Likewise, the effect of CORT is enhanced by the mineralocorticoid at level of its receptor (MR). This receptor has the same affinity for P4 and ALD, in a way that when it joins to P4, which is a competitive ALD inhibitor, it antagonizes its effects, inducing a transient natriuretic effect [48]. This initial natriuretic state induced by P4 triggers the compensatory secretion of ALD pathway SRA [49]. However, although natural glucocorticoids have some mineralocorticoid activity, this has minimal impact at physiological doses [50].

The ovulatory peak of ALD in mares confirms the results of previous research in women, related to estrogen-induced AOGEN synthesis [20], hemodynamic changes, alterations in local sympathetic activity, changes in Na^+^ concentrations at the level of the dense macula [51] and ACTH [52]. Previous studies in Spanish Purebred mares revealed that simultaneous increase in renin and angiotensin II suggests that the release of ALD represents the activity marked by the SRA at ovulation time [14]. In the present study, the correlations between ACTH and ALD were more consistent than with CORT, which indicates that ALD is mostly dependent on ACTH than CORT values.

After ovulation, ACTH and CORT concentrations decrease in mares during the first three and four days, although they significantly increase in day +5, simultaneously with the ALD. These results could indicate the dependence of both hormones as the luteal period progresses, since in women, an increase in ACTH might contribute to the peak of ALD [18]. This increase in ALD confirms the results obtained in women [18,53]. On the contrary, in other studies carried out in women [54] and female dogs [34] the HPA activity and circulating ACTH and CORT decreases during diestrus.

### 4.4. Electrolytes Balance and Endocrine Modulation in Different Species and in Mare

The concentrations of Na^+^ and Cl^−^ showed a significant increase on ovulation day, without other modifications. Nerveless, in the mare this trend results controversy. Two previous studies in mares by Kinslow et al. [21] and Collins et al. [55] showed large increases in the volumetric urinary excretion of Na^+^ in samples collected during the period from 2 days preceding to 4 days following estrus, compared to the rest of the cycle. Positive correlations between P4 and Na^+^, K^+^ and C1^−^ and between E2 and C1^−^ suggested that ovarian steroid hormones may exert an influence on urinary excretion of electrolytes in the mare. Indeed, the estrogen peak experiences a biphasic pattern over the urinary excretion of Na^+^, favoring the initial loss, followed by a retention period later, as occurs in sheep at the beginning of the luteal phase [56]. Therefore, the retention of Na^+^ that takes place during the luteal phase, represents a period directly related to the secretion of P4 and ALD [57]. P4 is generally considered to be a “Na^+^—losing” steroid or an “ALD antagonist” and it influences the metabolism of both Na^+^ and K^+^ in women [58].

The binding of free ALD to the MR receptor in the cytosol of epithelial cells of human, principally in the kidney, controls K^+^ homeostasis and maintains normal intravascular volume by increasing intestinal and renal Na^+^ and Cl^−^ absorption and reabsorption, respectively [22]. In Spanish Purebred mares, these physiological facts, related to the elevation of natremia during the follicular and luteal phase of the cycle, based on adrenal ALD secretion, were not possible to explain. However, as mentioned in the study by Szmuilowicz et al. [18], the increase in ALD in the luteal phase occurred only in women subjected to a high sodium balance, but not in those with a low balance, suggesting that the ALD is much more dependent on this balance than on hormonal status. However, this sodium balance was not considered in this study.

Moreover, K^+^ experienced a significant peak the day after ovulation with respect to previous days. These results partially confirm those obtained in sheep [56] and women during the luteal phase [59], although in this last study statistical significance was not reached, while it may decrease or not manifest oneself [60]. This evidence is based on physiological facts, such as the decrease in the urinary excretion of K^+^ that takes place during the ovulatory period in women [60]. These effects seem controversial, since ALD causes the redistribution of K^+^ from extracellular to intracellular compartments and stimulates tubular K^+^ secretion by the tubular cells. The tubular cells in the adrenal cortex responsible for secreting ALD are sensitive to the extracellular concentration of K^+^ surrounding them [61]. In the present study, the concentrations of Cl^−^ experienced two significant peaks; the first at the day of ovulation, and the second, at 3 days post-ovulation. However, although Cl^−^ correlates closely with P4 concentrations during the luteal period in women [57], these events were not confirmed in mares [55]. In cows, Cl^−^ levels correlate positively with the diameter of the follicle [62] and with E2 concentrations in buffalo [63]. According to Hugentobler et al. [64], metabolic changes at the systemic level affected the follicular fluid, as soon as the electrolyte balance could be closely related to the quality of the oocyte and GCs. In the authors’ opinion, endocrine differences in different species may be the source of some variations since a substantial body of evidence is already represented by the lack correlation between ALD and electrolytes.

In summary, in Spanish Purebred mares, along the natural ovulatory period, an activation of the HPA axis occurs, characterized by simultaneous increase of ACTH, CORT, and ALD concentrations. Hence, it is possible to presume that the pituitary adrenocortical response and ALD may be involved in the ovulatory mechanisms, without a direct relation with electrolyte pattern.

## Figures and Tables

**Table 1:** Electrolyte concentrations (mean ± SD) in cycling mares (n = 23) from day −5 pre-ovulation to day 5 post-ovulation.

	Pre-Ovulatory Days					Ovulation	Post-Ovulatory Days				
Days of Cycle	−5	−4	−3	−2	−1	0	+1	+2	+3	+4	+5
Na^+^ (mmol/L)	142.1 ± 0.33	141.1 ± 1.57	139.9 ± 2.47	141.3 ± 1.97	138.9 ± 0.99	142.2 ± 3.28 ^a,b^	141.8 ± 6.11	141.2 ± 3.27	143.2 ± 6.14	141.2 ± 4.79	140.5 ± 2.41
K^+^ (mmol/L)	4.18 ± 0.54	3.75 ± 0.56	4.29 ± 0.21	4.03 ± 0.15	3.88 ± 0.26	4.16 ± 0.26	4.51 ± 0.57 ^c^	4.35 ± 0.46	4.08 ± 0.71	4.30 ± 0.73	3.91 ± 0.74
Cl^−^ (mmol/L)	105.6 ± 0.92	104.9 ± 0.55	104.9 ± 1.96	105.2 ± 1.35	104.5 ± 1.28	107.4 ± 1.44 ^a,b^	106.0 ± 4.22	106.2 ± 2.77	108.8 ± 5.80	106.3 ± 3.95	104.4 ± 2.62

Different superscripts indicate significant differences between the ovulation day: a = vs. day −4; b = vs. day +5; c = vs. day −1 (*p* < 0.05).

## Data Availability

The datasets used and analyzed during the current study are available from the correspondent authors on reasonable request.
